# Pre-treatment With PLGA/Silibinin Nanoparticles Mitigates Dacarbazine-Induced Hepatotoxicity

**DOI:** 10.3389/fbioe.2020.00495

**Published:** 2020-06-26

**Authors:** Mikhail Durymanov, Anastasia Permyakova, Joshua Reineke

**Affiliations:** ^1^Department of Pharmaceutical Sciences, College of Pharmacy and Allied Health Professions, South Dakota State University, Brookings, SD, United States; ^2^Moscow Institute of Physics and Technology, Dolgoprudny, Russia

**Keywords:** drug-induced liver injury, hepatoprotection, melanoma, nanoformulation, drug combination

## Abstract

Drug-induced hepatotoxicity is one of the major barriers limiting application of current pharmaceuticals as well as clinical translation of novel and perspective drugs. In this context, numerous hepatoprotective molecules have been proposed to prevent or mitigate drug-induced hepatotoxicity. To date, silibinin (SBN) is a one the most studied hepatoprotective plant-derived agents for prevention/alleviation of drug-induced liver injury. Hepatoprotective mechanisms of SBN include scavenging of free radicals, upregulation of detoxifying enzymes *via* Nrf2 activation and inhibition of inflammatory activation of resident macrophages. However, low solubility of this phytochemical in water prevents its intravenous administration and constrains its bioavailability and efficacy. Here, we developed SBN-loaded poly(lactic-co-glycolic) acid (PLGA)-based nanoparticles for intravenous administration aiming at mitigation of drug-induced hepatotoxicity. Obtained nanoparticles demonstrated a slow drug release profile *in vitro* and caused upregulation of antioxidant and phase II enzymes in AML12 hepatocytes including superoxide dismutase 2, glutathione-S-transferase P1, and glutathione-reductase. Intravenous administration of PLGA nanoparticles to mice led to their fast liver accumulation. *In vivo* analysis of hepatoprotective effects of PLGA/SBN nanoparticles was carried out on melanoma tumor-bearing syngeneic mouse model treated with the antineoplastic drug dacarbazine (DTIC), which often causes severe hepatotoxicity including development of veno-occlusive disease. It was found that PLGA/SBN caused effective induction of detoxifying liver enzymes. Moreover, pre-treatment with PLGA/SBN nanoparticles reduced elevated transaminase and bilirubin levels in blood, caspase 3 activation, and morphological histology changes in liver tissue upon DTIC treatment. Treatment with PLGA/SBN nanoparticles did not interfere with therapeutic efficacy of DTIC.

## Introduction

Drug-induced liver injury remains a serious problem during pharmaceutical treatment of different diseases including cancer. Antitumor chemotherapy often leads to hepatotoxic side effects, which can result in discontinuation of the selected drug (King and Perry, 2001; Grigorian and O'Brien, 2014; Björnsson, 2016; Vincenzi et al., 2016). Some of the anticancer therapeutics including DTIC, flutamide and gemtuzumab belong to the FDA-approved drugs with a “black box” warning for hepatotoxicity (Guengerich, 2011). Among them, DTIC is a very effective alkylating cytotoxic agent generally prescribed for the treatment of malignant melanoma, soft tissues sarcoma, classical Hodgkin's and non-Hodgkin's lymphoma (Marchesi et al., 2007). In spite of high therapeutic efficacy, numerous studies report hepatotoxic adverse effects related with DTIC treatment including elevated liver enzymes and hepatic vascular toxicity (Erichsen and Jönsson, 1984; McClay et al., 1987; Ceci et al., 1988; Vincenzi et al., 2016). In part, hepatotoxic effects of DTIC are related with inhibition of DNA, RNA and protein synthesis due to its alkylating activity (Vincenzi et al., 2016). However, the main contribution to liver injury is associated with generation of the reactive oxygen species after DTIC treatment (Pourahmad et al., 2009).

Silibinin (SBN), the active compound in milk thistle, is a promising candidate to reduce drug-related hepatotoxicity, in which efficacy was confirmed in two recent clinical trials (Ladas et al., 2010; Gu et al., 2015). Although these clinical trials have indicated some extent of success, the treatment in both cases included high doses during long terms to achieve hepatoprotective effect. Numerous studies indicate multifaceted hepatoprotective effects of SBN. First, SBN promotes hepatocyte RNA polymerase I resulting in an increased synthesis rate of both structural and functional proteins (Sonnenbichler et al., 1976). Second, SBN increases expression of nuclear factor erythroid 2-related factor 2 (Nrf2) (Mehrab-Mohseni et al., 2011), responsible for activation of an antioxidant response and upregulation of protective enzymes such as NAD(P)H:quinone oxidoreductase 1 (NQO1), glutathione reductase, and glutathione S-transferases (GSTs) (Ma, 2013). Moreover, SBN is able to act as a free radical scavenger, providing an additional contribution to defense from oxidative stress (Muriel and Mourelle, 1990). Next, SBN stabilizes cellular membranes of hepatocytes and makes them more resistant to osmotic-stress-induced damage (Ramellini and Meldolesi, 1976). Finally, SBN is a strong inhibitor of leukotrienes and proinflammatory transcription factors like nuclear factor kappa B (NF-κB) (Dehmlow et al., 1996; Saliou et al., 1998; Hsiang et al., 2015) that explain its protective effect in an animal model of T cell-dependent liver injury (Schümann et al., 2003). Based on these hepatoprotective mechanisms, SBN is a reasonable agent for mitigation of liver injury caused by drugs producing free radicals and showing a hepatocellular pattern of toxicity. It should be noted that low aqueous solubility of this phytochemical and very fast elimination (<1 h) from the blood and different tissues including liver tissue (Christodoulou et al., 2015) strongly affects its bioavailability. On the other hand, nanoparticle-mediated delivery of SBN by intravenous administration leads to its improved pharmacokinetics in the liver compared with free (non-encapsulated) SBN (Jia et al., 2010).

Here, we aimed to test whether intravenous injection of clinically relevant SBN-loaded nanoparticles enables effective prevention/mitigation of DTIC-related hepatotoxicity. To achieve improved accumulation in the liver, SBN was encapsulated into poly(lactide-co-glycolide) (PLGA) biodegradable nanoparticles. In a syngeneic model of melanoma tumor-bearing mice we showed that pre-treatment with an intravenous PLGA/SBN formulation significantly reduced DTIC-induced liver toxicity without therapeutic efficacy interference of chemotherapy.

## Materials and Methods

### PLGA/SBN Nanoparticle Fabrication and Characterization

Nanoparticles loaded with SBN were prepared by a single emulsion-solvent evaporation technique. Briefly, 40 mg of Resomer^®^ RG 502 H poly(D,L-lactide-*co*-glycolide) (Evonik) and 4 mg of SBN (Cayman Chemicals, Ann Arbor, MI) were solubilized in 2 mL of acetone and homogenized for 5 min. The prepared solution was slowly added to 2% poly(vinylalcohol) aqueous solution. The mixture was kept under stirring (500 rpm) overnight at room temperature to evaporate organic phase. The NPs were collected by centrifugation at 20,000 × g for 15 min at 4°C and after removal of the supernatant (without disturbing the pellet) resuspended in 10 mL of deionized water by vortexing and collected by centrifugation again. After three washing cycles the purified nanoparticles were freeze-dried for 48 h. The purified nanoparticles were lyophilized for 48 h. Blank PLGA nanoparticles were made by the same method but without addition of SBN.

The quantification of SBN loading and release profile was performed using an HPLC Waters system (Waters Corporation, Milford, MA), equipped with a Phenomenex Luna C18 column (5 μm, 4.6 mm × 150 mm), an isocratic pump, a degasser, an autosampler, a UV/vis detector (Waters 2998 Photodiode Array Detector), and data processing software (Breeze version 3.30 SPA). The mobile phase containing 50% (v/v) acetonitrile in 0.05% acetic acid aqueous solution was delivered with a flow rate of 0.5 mL min^−1^. To measure SBN loading content, freeze-dried PLGA/SBN nanoparticles were dissolved in 30:70 (v/v) THF/acetonitrile mixture and filtered through 0.22 μm PVDF Fisher Syringe Filter. Chromatograms were monitored at 288 nm using an injection volume of 20 μL and temperature set at 25°C. The standard solutions, used for calibration curve, included SBN dissolved in 30:70 (v/v) THF/acetonitrile mixture with concentrations of 500, 250, 125, 62.5, 31.25, 15.62, 7.81, 3.91 μg mL^−1^. For release profile measurements, freeze-dried PLGA/SBN nanoparticles were resuspended in PBS at concentration of 1 mg per mL and incubated at 37°C under shaking. At designated time points (0.5, 1, 3 and 7 h, followed by 1, 2, 3.5, 4.5, 7 days), the suspension was centrifuged at 13,000 × g for 10 min and the supernatant was accurately harvested. The pellet was resuspended in fresh PBS in the original tube for further incubation maintaining near-sink conditions. Collected supernatants were subjected to quantitative analysis by HPLC. After collecting of the last supernatant, the pellet was used to analyze the residual SBN content. The release test was performed in triplicate.

Stability of PLGA/SBN nanoparticle formulation was evaluated after 1-month storage at 4°C by hydrodynamic diameters and polydispersity index measurements (DLS) and analysis of loading extent by HPLC.

PLGA nanoparticles loaded with rhodamine 6G (Acros Organics) were fabricated using the same technique. Rhodamine loading extent was measured using fluorescent spectroscopy at λ_ex_ = 530 nm and λ_em_ = 550 nm. The dye was extracted from nanoparticles by incubation in 30:70 (v/v) THF/acetonitrile mixture with concentration of 2 mg mL^−1^. Rhodamine content was determined according to calibration of rhodamine 6G standard solutions.

Hydrodynamic diameters, ζ-potentials and polydispersity index of generated PLGA nanoparticles were measured by dynamic light scattering using a Zetasizer Nano ZS (Malvern Instruments Ltd., Malvern, UK). The images of PLGA/SBN nanoparticles were obtained using FEI Quanta 450 scanning electron microscope.

### Cell Culture

Murine hepatocytes AML12 (ATCC^®^ CRL-2254™) were cultured in DMEM/F12 growth medium with 10% (v/v) of fetal bovine serum (HyClone), 1% penicillin/streptomycin mixture (HyClone) and ITS-G supplement (Gibco, Grand Island, NY). Murine melanoma B16 F1 cell line cells (ATCC^®^ CRL-6322™) were cultivated in RPMI-1640 growth medium with 10% (v/v) of fetal bovine serum (HyClone) containing 1% (v/v) of penicillin/streptomycin mixture. All cells lines were grown at 37 °? in a humidified 5% CO_2_ atmosphere.

### Preparation of Hepatocyte Cell Lysates for Western Blot Analysis

To determine induction of phase II and antioxidant enzymes, AML12 cells were seeded on a 6-well plate (10^5^ cells per well) and treated after 24 h with different formulations including SBN in DMSO (final concentration of SBN in growth medium is 100 μM), PLGA/SBN nanoparticles (containing 273 μg per mL of PLGA and 100 μM SBN at final concentration) and blank PLGA nanoparticles (273 μg per mL of PLGA at final concentration).

Formulations have been added 24 or 48 h before harvesting cells for preparation of cell lysates. Lysates of AML12 cells were obtained by addition of 300 μL of ice-cold lysis buffer (HEPES pH 7.5 containing 5% Triton X-100 and protease inhibitors) per well of a 6-well plate with cells, followed by 20-min shaking and collecting of supernatants upon centrifugation.

### GST Activity Measurement

For analysis of GST activity, we used AML12 cells seeded on a 6-well plate (10^5^ cells per well). The cells were cultured with SBN in DMSO (final concentration of SBN in growth medium is 100 μM) for 12, 36, or 48 h before harvesting. Cell lysates were prepared by incubation of AML12 cells with ice-cold phosphate-buffered saline (PBS, pH 7.4) containing 0.2 mM EDTA and 0.1% Triton X-100 for 30 min, followed by centrifugation (20,000 × g, 4°C, 20 min) and collecting of supernatants. Cell lysates of non-treated AML12 cells were used as a control.

GST activity was evaluated using 1-chloro-2,4-dinitrobenzene (CDNB) (Acros Organics, Morris Plains, NJ) assay. For the measurement of optical density at 340 nm, 1.4 mL of PBS, 100 μL of fresh cell lysate and 500 μL of cocktail (1 mM of CDNB and 2 mM of reduced glutathione in PBS) were mixed. Enzyme activity was calculated according to a formula:

$GST_{activity}=\frac{A_{340\;\left[\min^{-1}\right]}}{0.0096F\;\left[\mu mol^{-1}cm^{-1}\right]\times 1\;\left[cm\right]}÷\left(C_{protein}\left[\frac{mg}{mL}\right]\times V_{sample}\left[mL\right]\right)$, where *C*_*protein*_ is total protein concentration in a sample determined by Bradford assay and $A_{340\;}=\;\frac{\Delta A_{340\;sample}}{\Delta t}-\frac{\Delta A_{340\;blank}}{\Delta t}$. Kinetics of optical density change (*A*_340_) was determined using multiwell plate spectrophotometer SpectraMax M2 (Molecular Devices, Synnyvale, CA). All measurements were done in quadruplicate.

### MTT Assay

Evaluation of cytotoxicity of different formulations was carried out in 96-well plates using MTT assay. AML12 cells were seeded in a density of 5,000 cells per well 24 h before addition of SBN (in DMSO in non-toxic concentration range according to Supplementary Figure 1) or PLGA/SBN formulations in concentration range of SBN up to 0.2 mM. MTT assay was conducted after 24 h of incubation according to standard protocol. MTT reagent (3-(4,5-dimethylthiazol-2-yl)-2,5-diphenyltetrazolium bromide) (Acros Organics) was added to the growth medium at final concentration of 0.5 mg per mL, followed by incubation at 37°C for 4 h. After medium aspiration formazan crystals were solubilized in 100 μL of DMSO : ethanol (1:1, v/v) mixture. Optical density was measured using SpectraMax M2 spectrophotometer at 560 nm. The reference wavelength was 650 nm. All measurements were done in quadruplicate.

### Caspase 3/7 Activity Test

To determine protective effect of SBN formulations on AML12 cells under DTIC-induced cytotoxicity, we evaluated caspase 3/7 activity in treated cells. AML12 cells were seeded on 96-well plates with black walls in a density of 5,000 cells per well 24 h before addition of SBN or PLGA/SBN formulations in SBN concentration of 100 μM. Upon 24 h of incubation, the growth medium was changed with fresh medium with or without 0.5 mM DTIC (TCI America). After 48-h exposure to DTIC, caspase 3/7 activity was measured on a plate luminometer GloMax^®^-Multi detection system (Promega, Madison, WI) using Caspase-Glo^®^ 3/7 (Promega, Madison, WI) kit according to manufacturer's protocol. The measurements were done in quadruplicate.

### Establishment of Melanoma Tumors

Melanoma tumors were established by subcutaneous injection of 0.5 mln B16F1 cells into the right flank of 8-week-old male C57BL/6J mice (Jackson Laboratory, Ban Harbor, ME). Tumors were measured with calipers daily starting at day 3 post inoculation. Tumor volumes were calculated according to the formula V = (long axis × short axis^2^)/2. All animals were maintained in specific pathogen-free conditions with water and feed *ad libitum*. All animal experiments were approved by the South Dakota State University Institutional Animal Care and Use Committee.

### Biodistribution of PLGA/Rhodamine Nanoparticles in Tumor-Bearing Mice

When tumors have reached a volume of 200 mm^3^, PLGA/rhodamine nanoparticles were injected intravenously in a dose of 57 mg per kg of PLGA in 150 μL of PBS. Mice (3 animals per time point) were euthanized after 0.5 and 3 h post-injection and undergone transcardial perfusion. The organs were excised, weighed and homogenized in 10 mM Tris with 0.5% Triton X-100 in a ratio of 1 : 4.5 (w/v). After 1 h of incubation on ice, 30:70 (v/v) THF/acetonitrile mixture was added to tissue lysates in a THF/acetonitrile to lysate ratio of 4.5 : 5.5 (v/v), followed by 30-min incubation on ice, centrifugation (20,000 × g, 4°C, 20 min) and collecting of supernatants with extracted dye. For precise determination of rhodamine 6G concentration, calibration plots of increasing rhodamine concentrations in tissue lysates of different organs were built. Optical density of tissue lysates was measured in 96-well plates with black walls at λ_ex_ = 530 nm and λ_em_ = 550 nm using multiwell plate spectrophotometer SpectraMax M2.

### Treatment of Mice With Hepatoprotective Formulations for Determination of Liver Enzyme Expression

To determine expression level of antioxidant and phase II liver enzymes, mice were treated with different formulations. The first group received 500 μL of PBS intraperitoneally. The second group was intravenously injected with blank PLGA nanoparticles in PBS in a dose of 57 mg kg^−1^. The third group was treated with SBN in a dose of 10 mg kg^−1^ in 500 μL of PBS with 5% DMSO, injected intraperitoneally. Due to low solubility, SBN could not be administered intravenously. The fourth group of mice received intravenous injection of PLGA/SBN nanoparticles (10 mg kg^−1^ of SBN and 57 mg kg^−1^ of PLGA) in 150 μL of PBS. Upon 24 or 48 h, mice were euthanized, liver tissues were homogenized and lysed on ice in HEPES buffer (pH 7.5) containing 5% Triton X-100 and protease inhibitors (in a ratio tissue : lysis buffer of 1:4, w/w), followed by centrifugation (20,000 × g, 4°C, 20 min) and collecting of supernatants. For the analysis 3 animals per group and each time point were taken.

### Western Blot Analysis

Cell or tissue lysates with equal amounts of protein (40 μg) were mixed with loading dye, boiled for 5 min, separated on a denaturing 12.5-% SDS-polyacrylamide gel and transferred to Amersham™ Hybond™ 0.45 μm PVDF membrane (GE Healthcare, UK). The membrane was blocked in 5% dry milk or BSA in TBS buffer with 0.1% Tween (TBS-T) for 1 h and incubated overnight with antibodies against glutathione-S-transferase A3 (ab175246, Abcam), glutathione-S-transferase P1 (PA5-29601, Invitrogen), glutathione reductase (ab16801, Abcam), superoxide dismutase 2 (PA5-30604, Invitrogen), NAD(P)H quinone oxidoreductase 1 (PA5-19624, Invitrogen), pro-caspase 3 and active caspase 3 (ab13847, Abcam), or β-actin (4967, Cell Signaling) as a reference. Then, the membrane was washed twice with TBS-T and incubated with HRP-conjugated secondary antibody (ab6721, Abcam or sc-2005, Santa Cruz Biotechnology) at room temperature for 1 h, followed by several washings with TBS-T and deionized water. Protein bands were visualized by ChemiDoc XRS+ imaging system (Bio-Rad Laboratories, Hercules, CA) using chemiluminescence mode.

### DTIC Therapy of Tumor-Bearing Mice

All mice upon establishment of B16F1 tumors were randomly grouped (8 animals per group) for experiment with DTIC therapy. Pre-treatments with PBS or SBN formulations were performed on days 3, 6, and 9 after inoculation of cancer cells. PBS or SBN in a dose of 10 mg kg^−1^ in 500 μL of PBS with 5% DMSO were injected intraperitoneally, whereas PLGA/SBN nanoparticles (10 mg kg^−1^ of SBN and 57 mg kg^−1^ of PLGA) in 150 μL of PBS were administered intravenously. Treatments with antineoplastic drug DTIC or PBS (in control groups) were made on days 4, 7, and 10 after establishment of tumors. DTIC was dissolved in PBS and injected intraperitoneally in a dose of 110 mg per kg.

Following the experiment with DTIC therapy, 200 μL of blood were taken from the heart of mice under anesthesia. Blood serum was collected by centrifugation (20,000 × g, 4°C, 10 min) and immediately used for determination of alanine transaminase (ALT), aspartate transaminase (AST) and bilirubin levels using assay kits for determination of their content (all from Bioassay Systems, Hayward, CA), according to manufacturer's recommendations.

### Histological Assessment of Liver Samples

After withdrawal of blood samples, mice were transcardially perfused with 40 mL of PBS and 50 mL of 10-% buffered neutral formalin (Thermo Scientific) as a fixative. After 24-h incubation of liver tissues in formalin, they were embedded in paraffin wax and cut into 5 μm thickness slices, followed by staining with haematoxilyn-eosin. Tissues have been photographed using an inverted Olympus BX53F (Olympus Co., Tokyo, Japan) equipped with a UPlanApo 20 × /NA 0.70 objective lens.

### Statistical Analysis

Statistical analysis of results were performed using one-way analysis of variance (ANOVA) with *post-hoc* Tukey's test or Dunnett's *t*-test as indicated in the respective figures utilizing the appropriate test for the respective studies. Sample sizes are included within the respective methods section.

## Results

### PLGA-SBN Nanoparticle Characteristics and Cytotoxicity

**Figure 1:**
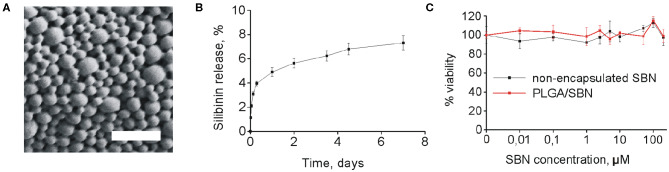
PLGA/SBN nanoparticle characterization and cytotoxicity. **(A)** SEM image of PLGA/SBN nanoparticles. Scale bar is 1 μm. **(B)** *In vitro* SBN release profile from PLGA/SBN nanoparticles in PBS at 37°C. **(C)** Viability of AML12 hepatocytes after 24 h of incubation with SBN or PLGA/SBN nanoparticles, determined by MTT assay.

SBN is considered to be a promising agent for activation of Nrf2/ARE pathway in terms of safety and efficacy (Abenavoli et al., 2018). However, its low solubility prevents its use for intravenous administration. Addressing this problem, we generated PLGA nanoparticles of 250 nm with SBN loading content of 15% (w/w) (Table 1 and Figure 1A). Release profile of SBN in PBS buffer at 37°C displayed very slow and gradual character. The data suggest that no significant SBN release or burst release are expected in the physiological environment. After a week of incubation only about 7% of SBN was released (Figure 1B) in near-sink conditions. The choice of a time frame for a release profile experiment is explained by PLGA degradation rate upon *in vivo* administration. It was found that after this term the major part (>70%) of injected PLGA was degraded and excreted from the organism (Mohammad and Reineke, 2013).

**Table 1:** Characterization of PLGA nanoparticle formulations^*^.

	**Hydrodynamic diameter, nm**	**PDI**	**Z-potential, mV**	**Loading content, %**	**Loading efficiency, %**
Blank PLGA	241.5 ± 9.2	0.199	−2.2 ± 0.2	-	-
PLGA/SBN	246.8 ± 13.5	0.197	−2.0 ± 0.2	15.2	52.3
PLGA/rhodamine 6G	252.2 ± 12.4	0.225	−1.8 ± 0.3	0.6	46.5

* *All values are shown as means ± SD*.

Obtained freeze-dried PLGA/SBN nanoparticles can be stably stored at 4°C for at least 1 month. By this period only a slight increase of polydispersity index was detected while no changes in nanoparticle size or loading extent was revealed (Supplementary Table 1).

According to literature data, SBN demonstrates protective effects in a concentration range of 25–500 μM in different cell lines (Surai, 2015). Therefore, we evaluated cytotoxicity of SBN and generated PLGA/SBN to AML12 hepatocytes in this concentration range (Figure 1C). It was found that neither formulations showed cytotoxic effects according to MTT assay.

### SBN Upregulates Antioxidant and Phase II Enzymes in AML12 Hepatocytes and Protects Them From DTIC-Induced Apoptosis

**Figure 2:**
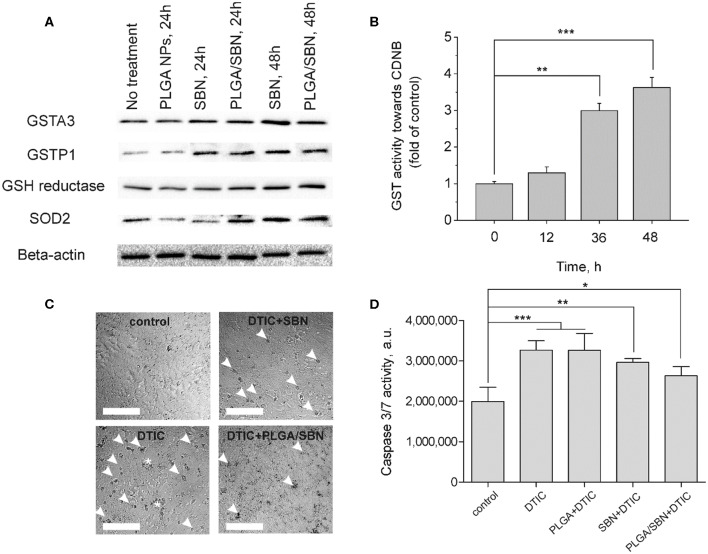
Induction of antioxidant response enzyme expression and hepatoprotective effect by SBN formulations in AML12 hepatocytes. **(A)** Western blotting analysis of liver enzyme expression in AML12 hepatocytes after treatment with blank PLGA nanoparticles, free SBN and PLGA/SBN nanoparticles. **(B)** GST activity kinetics in AML12 cells, incubated with 100 μM SBN, measured using CDNB assay. **(C)** Images of AML12 cells demonstrating antiapoptotic effect of free SBN or PLGA/SBN nanoparticle pre-treatments during incubation with 0.5 mM DTIC. White arrowheads point out single apoptotic cells, whereas white stars denote aggregations of apoptotic cells. Scale bar is 100 μm. **(D)** Reduction of caspase 3/7 activation in pre-treated with SBN-containing formulations AML12 cells during incubation with DTIC. All values are shown as means ± SD. ^*^P < 0.05, ^**^P < 0.01, ^***^P < 0.001 (one-way ANOVA followed by a *post-hoc* Dunnett's *t*-test) for **(B,D)**.

One of the key mechanism of SBN is its ability to activate Nrf2/ARE pathway in hepatocytes (Mehrab-Mohseni et al., 2011; Au et al., 2013; Surai, 2015). As far as we aimed to determine whether SBN pre-treatment may prevent or mitigate drug-induced liver injury, we analyzed how different SBN formulations affect expression of Nrf2-driven enzymes in AML12 hepatocytes. Among them, we evaluated the expression levels of glutathione reductase and glutathione-S-transferases A3 (constantly expressed) and P1 (inducible). Furthermore, we analyzed the change of SOD2 expression upon treatment with SBN formulations. Although SOD2 expression is not directly Nrf2-driven, this enzyme plays an important role in antioxidant defense. It has been shown earlier that SOD2 was upregulated by SBN treatment in HepG2 cells (Hsiang et al., 2015). It turned out that both free SBN and PLGA/SBN enhanced production of GSTP1, glutathione reductase and SOD2 reaching a maximum at 48 h of exposure (Figure 2A). Measurement of glutathione-S-transferase (all isoforms) activity also indicated maximal values at 48 h (Figure 2B) of incubation with SBN that follows Western blotting data.

Next, we evaluated, whether pre-treatment of AML12 hepatocytes with free SBN or PLGA/SBN can mitigate cytotoxicity induced by an anticancer drug DTIC. It was found that both pre-treatment with free SBN or PLGA/SBN nanoparticles efficiently decreased the number of apoptotic cells according to caspase 3/7 activity assay upon incubation with DTIC (Figures 2C,D) with PLGA/SBN having a larger effect. Probably, better efficacy of PLGA/SBN formulation might be attributed to encapsulated SBN, which remained within cells after growth medium change and addition of DTIC. The released SBN could be involved in scavenging of free radicals and ROS resulting in prevention of apoptosis in an additive to Nrf2 activation manner, whereas in cells, pre-treated with free SBN, it may be completely removed by change of growth medium and could not contribute to deactivation of reactive molecules at the same extent.

### PLGA Nanoparticle Biodistribution and Upregulation of Phase II Enzymes by SBN-Containing Formulations

**Figure 3:**
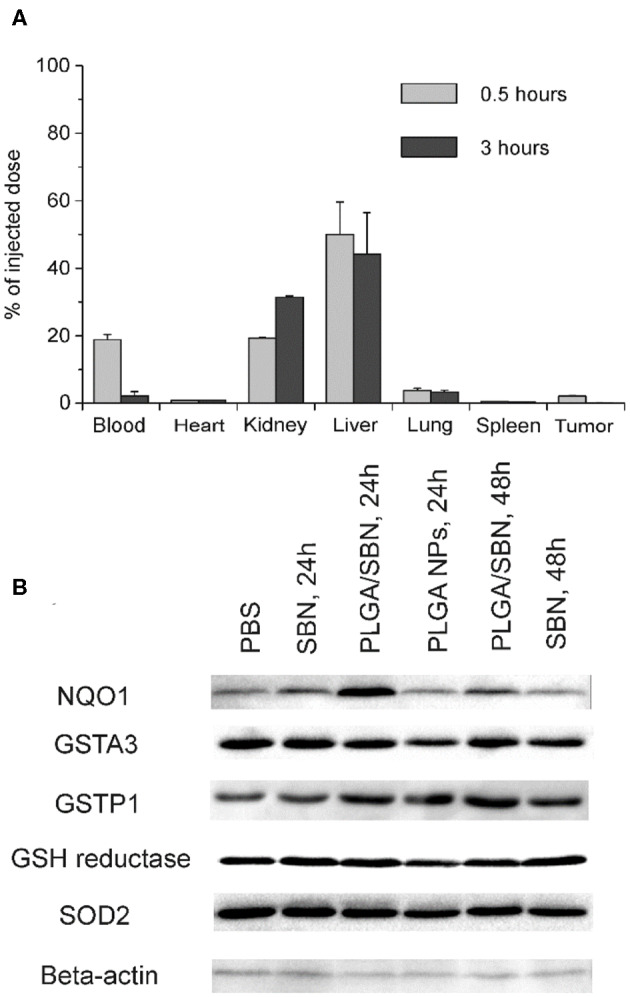
PLGA nanoparticle biodistribution and upregulation of phase II enzymes by SBN-containing formulations. **(A)** Analysis of PLGA/rhodamine nanoparticle biodistribution in different tissues of B16F1 tumor-bearing mice upon intravenous administration. All values are shown as means ± SD. **(B)** Expression levels of phase II and antioxidant enzymes in liver tissue of mice treated with SBN formulations, determined by Western blotting analysis.

We aimed to study the hepatoprotective effect of SBN formulations on a syngeneic melanoma mouse model of DTIC-induced liver injury. Since DTIC is a commonly used anticancer drug against melanoma, a particular interest was to explore the possible influence of SBN pre-treatment on the therapeutic effect of DTIC. In this context, we selected B16F1 tumor-bearing mice as a relevant model. It has been shown in previous studies that unmodified PLGA nanoparticles readily accumulate in the liver upon intravenous administration (Mohammad and Reineke, 2013). To validate high liver uptake of PLGA/SBN nanoparticles, we generated rhodamine-labeled particles with very similar physicochemical properties (Table 1) using the same fabrication method. We found fast PLGA/rhodamine nanoparticle deposition in the liver of tumor-bearing mice reaching up to 50% of intravenously injected dose within a half-hour. Of interest, only a small amount of nanoparticles accumulated in the tumor, followed by an almost complete wash-out during the next 2.5 h (Figure 3A). Considering this result in light of PLGA/SBN nanoparticle biodistribution indicates that PLGA-encapsulated SBN will be deposited in the liver at high extent and demonstrate low accumulation in the tumor. The advantage of SBN encapsulation for its superior accumulation in the liver in comparison with free SBN has been demonstrated earlier in a pharmacokinetic study (Jia et al., 2010). However, hepatoprotective effects of free SBN and intravenously administered encapsulated SBN formulations have not been studied. To analyze this, we compared the levels of phase II and antioxidant enzymes after pre-treatment with SBN formulations. It turned out that expression of NAD(P)H quinone oxidoreductase-1 (NQO1), one of the most responsive Nrf2 activation contributors to intracellular redox-potential, was significantly higher in the group treated with PLGA/SBN nanoparticles, whereas treatment with free SBN only slightly increased NQO1 expression in the liver (Figure 3B). Moreover, PLGA/SBN-treated group showed enhanced GSTP1 expression in comparison with free SBN-treated group or non-treated control group. Interestingly, no obvious alteration in SOD2 or GSH-reductase expression have been observed *in vivo* upon treatment with SBN formulations.

### Treatment With PLGA/SBN Nanoparticles Does Not Affect Therapeutic Effect of DTIC

**Figure 4:**
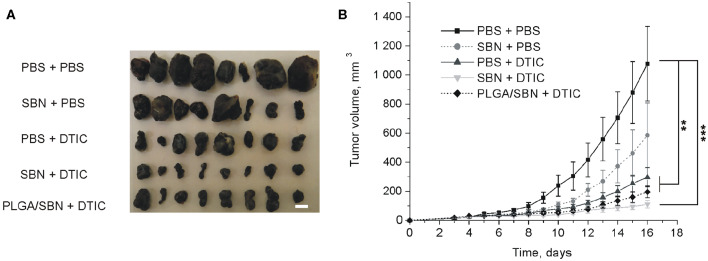
The impact of SBN-containing hepatoprotective formulation on anticancer therapeutic effect of DTIC. **(A)** Images of excised B16F1 tumors from different groups of mice. Scale bar is 5 mm. **(B)** Kinetics of tumor growth. Pre-treatments with PBS either SBN formulations were performed on the days 3, 6, and 9 after inoculation of cancer cells. Treatments with antineoplastic drug DTIC or PBS (in control groups) were made on the days 4, 7, and 10. All values are shown as means ± SEM. ^**^P < 0.01, ^***^P < 0.001 (one-way ANOVA followed by a *post-hoc* Tukey's test).

To study the hepatoprotective effect of PLGA/SBN nanoparticles, we carried out an experiment with therapy of syngeneic melanoma tumor-bearing mice treated with DTIC. The experiment included five groups of animals, which were pre-treated and treated with two formulations. A control group was treated twice with PBS (PBS + PBS). The second group was pre-treated with PBS and then treated with DTIC (PBS + DTIC) to demonstrate the therapeutic effect of DTIC without pre-treatment with hepatoprotectors. Two other experimental groups, SBN + DTIC and PLGA/SBN + DTIC, were pre-treated with SBN hepatoprotective formulations followed by DTIC treatment. Finally, the last group was pre-treated with free SBN, and treated later with PBS (SBN + PBS). This group was chosen as a control group of possible influence of SBN on tumor growth. It was found that SBN itself does slightly inhibit B16F1 tumor growth even without DTIC, although this effect was not statistically significant (Figures 4A,B). In contrast, all DTIC-treated groups demonstrated much slower increase of tumor volume as compared with control group. Interestingly, SBN + DTIC treatment showed slightly better but not statistically significant therapeutic effect than PBS + DTIC and PLGA/SBN + DTIC groups (Figures 4A,B). Such an interesting observation might be a result of previously reported anti-cancer effect of SBN (Li et al., 2010) and its higher bioavailability to tumor tissue as compared with encapsulated SBN, because according to our biodistribution study PLGA nanoparticles demonstrate low retention and fast wash-out from B16F1 tumors upon intravenous administration (Figure 3A).

### Treatment With Intravenous PLGA/SBN Nanoparticles Better Mitigates DTIC-Induced Liver Injury as Compared With Non-Encapsulated SBN

**Figure 5:**
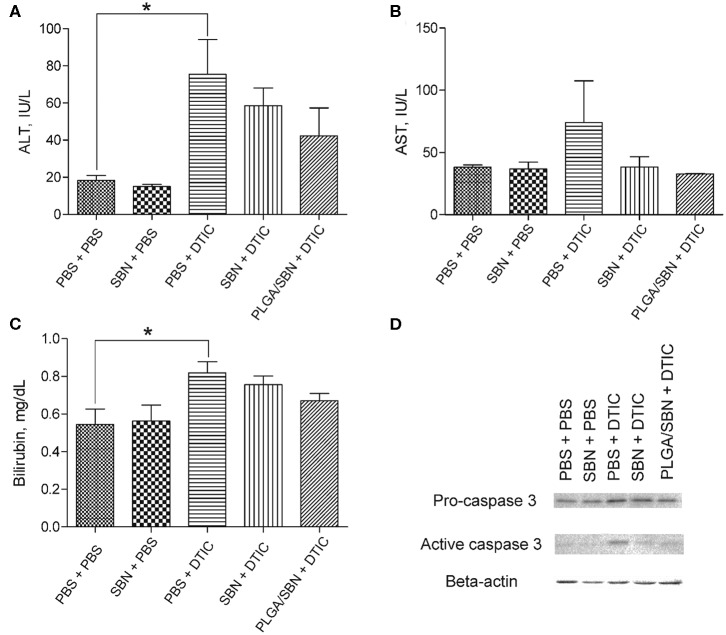
Hepatoprotective efficacy of SBN formulation pre-treatment. **(A)** ALT and **(B)** AST levels in blood serum of tumor-bearing mice after experiment with DTIC therapy. **(C)** Bilirubin content in blood serum. **(D)** Pro-caspase 3 and active caspase 3 levels in livers of treated tumor-bearing mice determined by Western blot analysis. All values are shown as means ± SD. ^*^P < 0.05 for **(A,C)** (one-way ANOVA followed by a *post-hoc* Dunnett's *t*-test).

After the therapeutic experiment with DTIC the mice were euthanized. Blood and liver tissues were analyzed in order to evaluate hepatoprotective effect of SBN formulations. We found elevated levels of ALT, AST and bilirubin in blood serum upon DTIC treatment. Pre-treatment with both free SBN and PLGA/SBN formulations decreased the levels of ALT, AST and bilirubin (Figures 5A–C). Although not significant, pre-treatment with PLGA/SBN nanoparticles led to much stronger reduction of the measured markers of liver injury. Besides the blood samples, we evaluated the level of active caspase 3 level in liver lysates, indicating apoptotic processes in liver tissue (Figure 5D). Caspase-3 is a cysteine protease, which mediates both apoptotic and necrotic cell death. Activation of this enzyme in a liver was detected in numerous types of hepatotoxicity, induced by pharmaceuticals (Chen et al., 2011), ethanol (Zhou et al., 2001) and viral infections (Bantel et al., 2001). It was found that both SBN formulations inhibited apoptotic processes in the liver to a similar extent (Figure 5D).

**Figure 6:**
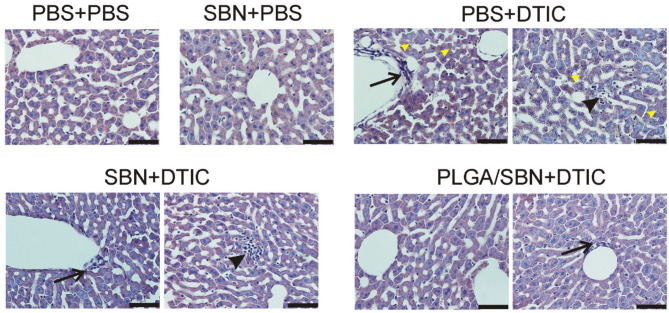
Morphology of liver tissue after chemotherapy with DTIC. Tissue slices of 5 μm thickness were stained with haematoxilyn-eosin. Black arrows denote mononuclear infiltration of sinusoids; black arrowheads denote necrotic foci; yellow arrowheads point to mild cholestasis. Scale bar is 50 μm.

We also performed an analysis of liver tissue histology in all groups of animals. We found in the group of PBS + DTIC some morphological changes associated with hepatotoxicity. First, we observed numerous cases of focal hepatocellular necrotic foci (Figure 6). Additionally, subendothelial infiltration with immune cells was revealed in number of liver sinusoids that might be the first prerequisite of sinusoid obstructive disease. Finally, DTIC treatment caused development of mild microvesicular steatosis in liver tissue. Pre-treatment with free SBN completely abrogated steatosis, although the cases of focal necrosis and mononuclear infiltrations of sinusoids were still present (Figure 6). At the same time, only a few sinusoids with signs of vascular injury were found in liver tissue samples from PLGA/SBN + DTIC group and no evidence of focal necrosis were found across all histological sections.

The intravenous PLGA/SBN nanoparticles had equivalent or better hepatotoxicity mitigation compared to free SBN as measured by ALT, AST, bilirubin, caspase-3, and liver histological morphology changes. This result is significant as the poor water solubility of SBN would otherwise limit it use in an intravenous formulation.

## Discussion

Drug-induced liver injury is a serious side effect associated with numerous medications, which can lead to treatment cessation or even patient deaths. Though drug-induced hepatotoxicity often has idiosyncratic character, related with polymorphisms, the mechanisms underlying liver damage are mostly associated with production of free radicals, oxidative stress and inflammatory reactions (Kaplowitz, 2005). In this context, the use of a hepatoprotective agent with pleiotropic effects such as SBN in parallel with a medication with high hepatotoxicity occurrence might prevent/mitigate the adverse effects. SBN treatment was shown to alleviate liver injury related with doxorubicin (Patel et al., 2010; Rašković et al., 2011), acetaminophen (Bektur et al., 2016), and cisplatin (Mansour et al., 2006) treatments. Moreover, two clinical studies indicated hepatoprotective effect of SBN for tuberculosis patients treated with conventional 2HREZ (S)/4HR_3_ (Gu et al., 2015) and in childhood acute lymphoblastic leukemia patients upon therapy with methotrexate (Ladas et al., 2010). In both preclinical and clinical studies SBN treatment regimen was long-term and included high doses. Such characteristics of SBN treatment are due to its very low oral bioavailability (0.95 %) (Wu et al., 2007) originating from poor aqueous solubility (<0.04 mg per mL) and fast elimination of this drug from the blood and different tissues (Wu et al., 2007; Jia et al., 2010; Christodoulou et al., 2015). In this context, a few studies aiming at SBN encapsulation in order to improve its bioavailability were performed. Two studies are focused on development of SBN-containing solid (Yang et al., 2013) or polymer-based (Guhagarkar et al., 2015) nanoparticles with enhanced oral bioavailability. In both cases, developed formulations displayed improved hepatoprotective efficacy in comparison with conventional SBN formulation on a CCl_4_ rat model, though high-dose and long-term treatment modalities were still employed. In another study the authors developed polymeric SBN-loaded nanoparticles for intraperitoneal administration. These nanoparticles also demonstrated superior hepatoprotective properties in acetaminophen-induced liver injury model (Das et al., 2011). However, intraperitoneal administration is not common for treatment in human patients. All the clinical and pre-clinical hepatoprotection studies with SBN utilized large doses and suffered from low bioavailability, yet none have explored intravenous administration due to the poor water solubility of SBN. A pharmacokinetics study revealed that encapsulation of SBN into lipid nanoparticles followed by intravenous injection significantly enhances liver tissue bioavailability of SBN as compared with free drug administration (Jia et al., 2010).

Here, we studied the hepatoprotective effect of PLGA-encapsulated SBN during anticancer therapy of melanoma tumors with DTIC, which often causes hepatotoxicity (Erichsen and Jönsson, 1984; McClay et al., 1987; Ceci et al., 1988; Vincenzi et al., 2016). The choice of PLGA as a carrier for SBN delivery is explained for several reasons. First, PLGA is a safe and biodegradable polymer approved by FDA for intravenous administration. Furthermore, unmodified PLGA-based nanoparticles tend to have fast uptake by liver macrophages and partly hepatocytes from circulation due to the hydrophobic nature of this polymer. Upon intravenous administration, almost 50% of the injected dose of PLGA nanoparticles accumulate in the liver in the first hours and undergo gradual digestion for more than 1 week (Mohammad and Reineke, 2013). For the reason of very slow release profile in PBS (Figure 1B), SBN is expected to be released from PLGA nanoparticles upon their degradation in Kupffer cells and distribute among adjacent hepatocytes and stellate cells resulting in elevated local tissue concentration as compared with free SBN, or encapsulated SBN administered orally or intraperitoneally. Thus, use of PLGA/SBN formulation for intravenous administration is expected to be more effective in prevention of drug-induced hepatotoxicity than administration of free SBN. We found that rhodamine-labeled PLGA-based nanoparticles immediately accumulated in the liver upon injection (Figure 3A) suggesting efficacy of PLGA nanoparticles for delivery of different loads, including hepatoprotective agents. Comparison of hepatoprotective properties of free SBN and PLGA/SBN nanoparticles has shown the benefit of encapsulation. First, PLGA/SBN more efficiently activated Nrf2 target gene NQO1 and upregulated GSTP1 in a liver than free SBN making hepatocytes more resistant to damaging agents such as DTIC. Second, pre-treatment with encapsulated SBN demonstrated stronger reduction of liver enzymes and bilirubin in blood serum caused by DTIC (Figures 5A–C). Finally, pre-treatment with PLGA/SBN better preserved normal liver tissue morphology upon therapy with DTIC (Figure 6).

An important focus of our study was to evaluate, whether SBN formulations affect anticancer therapy with DTIC. This interest is due to numerous reports of anti-cancer effects of SBN. Antitumor effect of SBN treatment alone or in combination with other agents has been shown on tumor models of breast cancer (Forghani et al., 2014), prostate cancer (Deep et al., 2008), lung cancer (Wu et al., 2016), and numerous *in vitro* studies (Li et al., 2010). Among intracellular targets, SBN inhibits STAT3, tyrosine kinase and NF-κB signaling pathways (Li et al., 2010). Our data indicated a tendency to inhibition of B16F1 tumor growth by free SBN (SBN + PBS group) (Figure 4). In combination of free SBN and DTIC anti-cancer effect seemed to have an additive character (SBN + DTIC group). As opposed to the liver, PLGA nanoparticles demonstrated limited accumulation and fast wash-out from tumor tissue (Figure 3A), that probably did not lead to SBN accumulation in tumor tissue. As a result, tumor growth kinetics in PBS + DTIC and PLGA/SBN + DTIC groups look very similar. However, Nrf2-activating property of SBN might have an unpredictable effect on tumor growth. Some studies indicated tumorigenic effect of Nrf2 activation, for example, in the case of pancreatic cancer (DeNicola et al., 2011; Hayes et al., 2015). In this context, accumulation of SBN in tumor tissue might be undesirable.

Thus, the use of PLGA/SBN nanoparticles for prevention/alleviation of hepatotoxicity during anticancer therapy demonstrated improved hepatoprotective efficacy and did not affect tumor growth as compared with free SBN, which interaction with tumor cells might have unpredictable consequences for cancer progression.

## Conclusion

We developed here an intravenous PLGA/SBN nanoparticle formulation showing a controlled release profile, enhanced induction of phase II enzymes, and improved protective properties in DTIC-induced hepatotoxicity as compared with free SBN. It should be emphasized that mitigation of DTIC-induced liver injury was achieved even after single injection of PLGA/SBN nanoparticles that probably occurs due to better bioavailability of encapsulated SBN for liver tissue.

## Data Availability Statement

The datasets generated for this study are available on request to the corresponding author.

## Ethics Statement

The animal study was reviewed and approved by Institutional Animal Care and Use Committee, South Dakota State University.

## Disclosure

All authors are listed as inventors on a provisional use patent related to the work presented here.

## Author Contributions

All authors contributed to the experimental design and project theory. MD and AP conducted all experiments. All authors contributed to the writing and editing of the manuscript.

## Conflict of Interest

The authors declare that the research was conducted in the absence of any commercial or financial relationships that could be construed as a potential conflict of interest.
