# Supplementary material for: Pre-treatment With PLGA/Silibinin Nanoparticles Mitigates Dacarbazine-Induced Hepatotoxicity
Source: Front Bioeng Biotechnol. 2020 Jun 26;8:495. doi: 10.3389/fbioe.2020.00495 (PMC7332747; doi:10.3389/fbioe.2020.00495)
Supplement: Supplementary file 1 [file Table_1.DOCX]

**Supplementary Table 1.** Characterization of PLGA/SBN nanoparticle formulation before and after one-month term of storage at 4 °C^*^.

|  | Hydrodynamic diameter, nm | PDI | Loading content, % |
| --- | --- | --- | --- |
| PLGA/SBN | 246.8±13.5 | 0.197 | 15.2±0.6 |
| PLGA/SBN (after one month) | 257.0±16.4 | 0.276 | 15.3±0.2 |

^*^All values are shown as means ± SD.





**Supplementary Figure 1.** Viability of AML12 hepatocytes after 24 hours of incubation with DMSO in a relevant for SBN dilution concentration range.
